# Annotations

**Published:** 1894-03-31

**Authors:** 


### Annotations.

### Tangier as a Health Resort.

Happy is the healthless modern who finds value for money in a health resort! But the other day a well-known public man returned from a fortnight's sojourn at a favourite continental refuge for the weary, sick in mind and body, and wishing he had never left England. The climate in that part of Europe, which, for obvious reasons, we do not particularise, he found little or no better than that of our own moist and misty island. Tangier, Mr. Ernest Hart tells us, is very much better than that. "To the professional man," he says, "escape for however short a holiday from the fogs, the darkness, the drizzle, and the various ferocities of a British winter, is a welcome respite and refreshment, a recreation of mind, and a renewal of life to the body." Such a short holiday may be spent in the winter at Tangier, on the westernmost point of Northern Africa. "For invalids, convalescents, health-seekers, and idlers alike," Mr. Hart assures us, "it has the charm of a climate of unsurpassed equality and moderation, equally delightful in summer as in winter, of a great wealth of sunlight, and of a picturesqueness which words are impotent to paint." Admirable this; the very place for a moisture-soaked Englishman. Infinitely is Tangier to be preferred to Cairo. That Egyptian city is said to be fast becoming a French town with an English climate, since the opening of the Suez Canal. Tangier is still a place of Moors and mosques, of distant hills and a rolling sea, with an old world freedom from European conventionalities and cares quite delightful to the jaded and overworked struggler with the ideas and strenuous practicalities of the western world. If Mr. Ernest Hart may be trusted on this question—and he may—a month at Tangier in the winter may result in a year of health.

### Brain Scavengers.

The researches of the modern physiologist promise to effect a complete revolution in the position of the scavenger, and in the estimation in which he is popularly held. Phagocytes, the scavengers which rush to a newly-inflicted wound and seize upon any dangerous microbes that may be entering by the breach, are now very familiar organisms, and as honourable in our eyes as they are familiar. What all of us wish is that they could be increased in numbers tenfold, and in energy a hundredfold within the body. Deiter's cells, the so-called "spider cells," which are developed in the neuroglia of the cerebral substance, and which have hitherto been regarded as a part of the connective tissue framework of the cerebral organ, are now affirmed by Dr. Bevan Lewis to have another function than that of merely holding in position the brain parenchyma Dr. Lewis considers that they have a true phagocytal function, in other words, that they are brain scavengers. The question is very ably discussed by Dr. Edwin Goodall, pathologist and assistant medical officer to the West Riding Asylum, in the February number of the *Journal of Pathology*. Dr. Goodall has made a large number of experiments on the brains of rabbits. He has established the fact that irritation of the cerebral cortex sufficient to set up inflammatory action of a severe kind leads to the final result of the production of a cicatricial tissue —to sclerosis in fact; and this is quite what one would expect holding the doctrine that Deiter's cells are histologically of a true connective tissue origin and character. What he has failed to find is evidence of a phagocytal function. Ferrocyanide of copper, which is of a brown colour, was injected into the experimental wounds of the cortex in a state of fine suspension in water, but never at any time was there evidence that any of it was taken up by the "spider cells." Dr. Bevan Lewis' theory is decidedly interesting, but it as decidedly lacks proof. Developmental as well as structural reasons would seem to almost demonstrate a negative. Nevertheless the inquiry ought to be pursued. The idea of a special organism for the scavenging of the brain, and consequently for the clarification of the intellect, is as stimulating as it is novel. The adequate scavenging of the general brain is undoubtedly one of the most urgent of all the necessities of civilisation.

### An Incompatible Mixture.

We have received a series of papers, one of which is a pamphlet entitled "Shipowners and Ships' Surgeons," by Charles Henry Leet; another is an advertisement of "Dr. Leet's Pills," and a third consists of Dr. Leet's recent testimonials from doctors and private patients. To the pamphlet on "Shipowners and Ships' Surgeons" no well-informed man can object. Undoubtedly ships' surgeons occupy an impotent and a miserable position. If it be a reasonable thing to put a window into a house, and then to brush the glass all over with black paint, then it may be a reasonable thing to appoint a fully qualified medical man to the surgeoncy of a ship, and to deny him any right whatever to do what he thinks best for the sanitation of the ship and the health of the passengers and crew. On this point there cannot be two opinions among honest and sensible people, and the sooner the Board of Trade puts an end to the present farce of medical supervision and authority on board passenger and mercantile ships the better it will be for the morals and manhood of the doctors as well as for the health of the passengers and crews. All this is obvious. But when Mr. Charles Leet mixes up this attempt at public duty with advertisements for pills and testimonials from patients, it does not require the intelligence of a medically instructed man to see that the public service is made a stalking-horse for the sale of pills. This is an absolutely incompatible mixture, and a grievous lapse from the honourable status of right professional conduct to the level of unworthy quackery. Mr. Leet is, or was, a Fellow of the College of Surgeons. We do not wish to say one harsh word about him. Probably he is by no means happy in his present way of life. We would suggest repentance to him and all such. If a man falls into a bad way, he need not remain in it. Undoubtedly the medical profession is as ready to forgive, and to receive again an erring brother as any other. Let Mr. Leet try what a return to honourable professional conduct will do for him! Of all quackeries the quackery of a legally qualified medical man is the most indefensible.

